# The prevalence of co-morbidities and their impact on physical activity in people with inflammatory rheumatic diseases compared with the general population: results from the UK Biobank

**DOI:** 10.1093/rheumatology/key224

**Published:** 2018-08-09

**Authors:** Michael J Cook, Eftychia Bellou, John Bowes, Jamie C Sergeant, Terence W O’Neill, Anne Barton, Suzanne M M Verstappen

**Affiliations:** 1 Arthritis Research UK Centre for Epidemiology, Division of Musculoskeletal & Dermatological Sciences, Manchester, UK; 2 Arthritis Research UK Centre for Genetics and Genomics, Division of Musculoskeletal & Dermatological Sciences, School of Biological Sciences, Faculty of Biology, Medicine and Health, The University of Manchester, Manchester Academic Health Science Centre, Manchester, UK; 3 Division of Population Health, Health Services Research & Primary Care, School of Health Sciences, Faculty of Biology, Medicine and Health, University of Manchester, UK; 4 NIHR Manchester Biomedical Research Centre, Central Manchester University Hospitals NHS Foundation Trust, Manchester Academic Health Science Centre, Manchester, UK

**Keywords:** rheumatic diseases, autoinflammatory diseases, co-morbidity, physical activity, population studies

## Abstract

**Objectives:**

To compare the prevalence and incidence of chronic co-morbidities in people with inflammatory rheumatic and musculoskeletal diseases (iRMDs), and to determine whether the prevalent co-morbidities are associated with physical activity levels in people with iRMDs and in those without iRMDs.

**Methods:**

Participants were recruited to the UK Biobank; a population-based cohort. Data were collected about demographics, physical activity, iRMDs (RA, PsA, AS, SLE) and other chronic conditions, including angina, myocardial infarction, stroke, hypertension, pulmonary disease, diabetes and depression. The standardized prevalence of co-morbidities in people with iRMDs was calculated. Cox regression was used to determine the relationship between the presence of an iRMD and an incident co-morbidity. The relationship between the presence (versus absence) of a (co-)morbidity and physical activity level (low, moderate, high) in people with iRMDs and in those without was assessed using multinomial logistic regression.

**Results:**

A total of 488 991 participants were included. The estimated prevalence of each co-morbidity was increased in participants with an iRMD, compared with in those without, particularly for stroke in participants with SLE (standardized morbidity ratio (95% CI), 4.9 (3.6, 6.6). Compared with people with no iRMD and no morbidity, the odds ratios (95% CI) for moderate physical activity were decreased for: no iRMD and morbidity, 0.87 (0.85, 0.89); iRMD and no co-morbidity, 0.71 (0.64, 0.80); and iRMD and co-morbidity, 0.58 (0.54, 0.63).

**Conclusion:**

Having a (co-)morbidity is associated with reduced physical activity in the general population, and to a greater extent in participants with an iRMD. Optimal management of both iRMDs and co-morbidities may help to reduce their impact on physical activity.

**Rheumatology key messages**

- The prevalence of all morbidities considered was increased in participants with RA compared with the general population.
- The prevalence of stroke was particularly increased in participants with SLE compared with the general population.
- (Co-)morbidity is associated with reduced physical activity, particularly in people with an inflammatory rheumatic and musculoskeletal disease.

## Introduction

Musculoskeletal diseases, including chronic rheumatic and musculoskeletal diseases (RMDs), are a major global burden, as measured by disability-adjusted life years [1, 2]. The most prevalent inflammatory RMDs are RA, PsA, AS and SLE, with estimated prevalence rates in European adults of 0.8% [3], 0.2% [4], 0.11% [5] and 97 cases per 100 000 population (0.1%) [6], respectively. Patients with these diseases have an increased risk of co-morbid conditions compared with the general population [7, 8], partly explained by the chronic inflammation. Most notably, the prevalence and incidence of cardiovascular disease is increased in people with inflammatory RMDs, and accounts for >40% of premature deaths [9–15]. Pulmonary disease [12, 13, 15–17], diabetes [13, 14] and depression [13, 15] are also more common in people with inflammatory RMDs. However, most studies investigating the prevalence of co-morbidities in people with inflammatory RMDs have been undertaken in disease-specific cohorts, and prevalence estimates of co-morbidities vary considerably between studies, partly due to differences in study design, population characteristics and disease ascertainment.

In a nested case–control study using primary care data from the Netherlands [7], patients with inflammatory arthritis were more likely to have cardiovascular disease (odds ratio (OR) 1.4, 95% CI 1.2, 1.5), respiratory disease (OR 1.5, 95% CI 1.3, 1.8) and depression (OR 1.2, 95% CI 1.0, 1.4), compared with age- and sex-matched controls without inflammatory arthritis. To gain a better understanding of multimorbidity in general populations, it is important to estimate rates of morbidity in one population using consistent methods for disease ascertainment.

Physical activity has many benefits for patients with inflammatory RMDs, including reducing disease activity and pain, increasing functional capacity and improving psychological health [18, 19], as well as potentially reducing the incidence of some co-morbidities, including cardiovascular disease, diabetes and osteoporosis [20]. There is some evidence that patients with inflammatory RMDs are less physically active than the general population [21–23]. However, the relationship between co-morbid diseases and physical activity in patients with different inflammatory RMDs has not been explored in detail. Such data are important for understanding the barriers to physical activity and highlighting the importance of effective co-morbidity screening and management in patients with inflammatory RMDs.

The primary objective of this study was to determine the prevalence and incidence of chronic co-morbidities in people with an inflammatory RMD, including RA, PsA, AS or SLE, compared with people without an inflammatory RMD in a large, national cohort from the UK (UK Biobank). Our secondary objective was to determine whether prevalent multi-morbidity is associated with physical activity level in people with an inflammatory RMD compared with those without these conditions.

## Methods

### Study design and participants

Over 0.5 million men and women aged 40–69 years were recruited to the UK Biobank cohort from primary care registers from 2007 to 2010 [24, 25]. Participants attended an assessment centre in the UK and completed a detailed questionnaire on a touch-screen computer about their lifestyle, including smoking status (never, past or current) and frequency of alcohol consumption (daily or almost daily, 2–3 times per week, 1–2 times per week, 1–3 times per month, special occasions only or never). Participants were subsequently interviewed by a trained research nurse to determine whether they had been diagnosed with any medical conditions and their medication use. Physical measurements were also taken, including blood pressure, weight and height, which were used to calculate BMI. The Townsend deprivation index was calculated for each participant immediately prior to recruitment to the UK Biobank based on the preceding national census output areas [26].

The UK Biobank study was approved by the North West Multi-Centre Research Ethics Committee, and all participants provided written informed consent. No additional ethical approval was required for this study.

### Physical activity

Physical activity was recorded using the International Physical Activity Questionnaire, a validated physical activity instrument [27]. Participants were asked about the number of days per week and the number of minutes per day they typically spend doing physical activity in bouts of ⩾10 min at three levels: walking, moderate (e.g. cycling at a normal pace) and vigorous (e.g. aerobics). Participants were categorized as having a low, moderate or high physical activity level based on the International Physical Activity Questionnaire algorithm [28]. We also considered the proportion of participants with and without an inflammatory RMD who carried out the World Health Organization (WHO)-recommended level of physical activity, which is at least 150 min of moderate-intensity physical activity, or at least 75 min of vigorous-intensity physical activity per week (in bouts of ⩾10 min) for adults [29].

### Morbidities

Participants were asked whether they had ever been told by a doctor that they had certain common medical conditions, including heart attack, angina, stroke, high blood pressure, diabetes (any type) and emphysema/chronic bronchitis. Other co-morbidities, including chronic obstructive pulmonary disease and depression were recorded during the interview with a research nurse. Morbidities were coded in the UK Biobank database using a hierarchical structure loosely based on International Classification of Diseases-10 (ICD-10) codes [30].

All data were collected at the UK Biobank baseline assessment. Participants were asked to self-report the age/year of diagnosis of their inflammatory RMD and/or co-morbidities. The retrospective, self-reported date of diagnosis of inflammatory RMD and co-morbidities was used to identify incident cases of co-morbidity occurring after the diagnosis of one of the inflammatory RMDs, and also the time (years) between the diagnosis of the inflammatory RMD and the diagnosis of the co-morbidity. Incident co-morbidities could not be identified for ∼1% of participants with an inflammatory RMD due to missing data. To assess the association between co-morbidities and physical activity, a modified functional co-morbidity index [31, 32] was calculated for each participant using self-reported morbidities. The functional co-morbidity index was developed by identifying co-morbidities that correlate with the physical function subscale of the 36-Item Short-Form Health Survey (SF-36) [31] (see Supplementary data, section *Construction of the functional co-morbidity index*, available at *Rheumatology* online). Furthermore, we classified each participant into one of four study groups, depending on whether they had any one of the four inflammatory RMDs being studied and whether their functional co-morbidity index was >0.

### Identifying participants with inflammatory RMDS

Participants answering yes to the question ‘Has a doctor ever told you that you have had any other serious medical conditions or disabilities?’ were asked the following question during the interview: ‘in the touch-screen questionnaire, you selected that you have been told by a doctor that you have other serious illnesses or disabilities. Could you now tell me what they are?’ Conditions were selected from a pre-specified list, including RA, PsA, AS and SLE, or entered as free text if they did not exist in the pre-specified list. Participants also reported their age at the time of diagnosis or the year of diagnosis. Participants who did not answer whether they had a chronic medical condition, who reported having non-specified arthritis, or who reported having more than one of the four inflammatory RMDs considered were excluded from the analyses (Supplementary Fig. S1, available at *Rheumatology* online).

### Medication

During the interview, details of medications being used at the time of the assessment were recorded by the research nurse, by selecting the drug name from a pre-specified list. Medications not included in the pre-specified list were entered as free text. We developed an algorithm to code free text medication data (see Supplementary data, section *Coding free text medication*, available at *Rheumatology* online). Medication data was used to determine the number of people who were using synthetic and biologic DMARDs (s/bDMARDs), and oral CSs.

### Statistical analyses

Descriptive statistics were used to summarize population characteristics. Results are presented as median and interquartile range for continuous variables, and number of participants (%) for categorical variables. Pairwise comparisons of the subject characteristics were performed for participants with each of the four individual inflammatory RMDs, compared with subjects without these inflammatory RMDs using the Mann–Whitney *U*-test for continuous variables and the χ^2^ test for categorical variables. A two-tailed *P *< 0.05 was considered statistically significant.

For each of the four inflammatory RMDs individually, indirect standardization was used to calculate the 5-year age band- and sex-adjusted prevalence and morbidity ratios (SMRs). The reference population (controls) comprised participants who did not have one of the inflammatory RMDs considered in our study. Sensitivity analyses were performed, only including participants having one of the inflammatory RMDs considered and also reporting to use s/bDMARDs.

To determine the risk of incident co-morbidities occurring after the diagnosis of an inflammatory RMD, participants with an inflammatory RMD were age- and sex-matched to four control participants. Cox regression models were used to determine the hazard ratio for the development of chronic co-morbidities compared with controls, adjusting for age and sex. The index date was the time of diagnosis of an inflammatory RMD; the same date was used for the matched controls. The proportional hazards assumption was assessed using the formal Schöenfeld’s residual test. Incidence and prevalence estimates based on <10 cases were not reported.

Multinomial logistic regression was used to determine the independent association between (co-)morbidity and physical activity level (low, moderate and high), adjusting for age, sex, smoking, alcohol consumption and BMI, by applying three different models. First, we investigated the association between physical activity level and co-morbidity (modified functional co-morbidity index >0) in participants with and without an inflammatory RMD (any one of the inflammatory RMDs studied as a single group). A dummy variable was generated to indicate four study groups: participants with no inflammatory RMD and no co-morbidity (referent group), participants with none of the four inflammatory RMDs and a co-morbidity, participants with one of the four inflammatory RMDs and no co-morbidity, and participants with one of the four inflammatory RMDs and a co-morbidity. Second, we looked at the association between physical activity level and individual co-morbid disease types in people with inflammatory RMDs. Finally, we looked at the association between physical activity level and the functional co-morbidity index (categorized as 0, 1–2, 3–4 and ⩾5) in people with one of the four inflammatory RMDs. The proportion of participants with and without one of the investigated inflammatory RMDs who carried out the WHO recommended amount of physical activity was compared using a χ^2^ test. All analyses were conducted using Stata V.13.1 (Stata Corp, College Station, Texas, USA).

## Results

Of the 502 643 participants recruited to UK Biobank, 488 991 were included in the present study (Supplementary Fig. S1, available at *Rheumatology* online). Differences in subject characteristics between participants included and excluded from the analyses are shown in Supplementary Table S1, available at *Rheumatology* online. Of the participants included, 480 998 (98.4%) had none of the four inflammatory RMDs, 5315 (1.1%) participants had RA, 865 (0.2%) had PsA, 1254 (0.3%) had AS and 559 (0.1%) had SLE (Table 1). The median (interquartile range) age of participants with RA, PsA, AS, SLE and no inflammatory RMD was 61.0 (55.0–65.0), 57.0 (51.0–62.0), 59.0 (52.0–63.0), 56.0 (49.0–62.0) and 58.0 (50.0–63.0) years, respectively. The proportion of participants with an inflammatory RMD using a DMARD varied from 48% in RA and PsA to 8% in AS. The proportion using oral CSs ranged from 4% in AS to 20% in SLE. The proportion of participants using a s/bDMARD or oral CSs in the control population was 0.4% and 1.1%, respectively (Table 1).

**Table 1:** Subject characteristics

	None of the study conditions (*N* = 480 998)		RA (*N* = 5315)		PsA (*N* = 865)		AS (*N* = 1254)		SLE (*N* = 559)	
	Median (IQR) or %	*n*	Median (IQR) or %	*n*	Median (IQR) or %	*n*	Median (IQR) or %	*n*	Median (IQR) or %	*n*
Age, years	58.0 (50.0–63.0)	480 998	61.0 (55.0–65.0)^a^	5315	57.0 (51.0–62.0)^a^	865	59.0 (52.0–63.0)^a^	1255	56.0 (49.0–62.0)^a^	560
Age at onset of rheumatic/musculoskeletal disease, years	—	—	48.3 (37.9–56.0)	5255	44.7 (35.0–52.2)	855	36.1 (25.5–46.9)	1242	42.0 (23.8–51.3)	555
Female	54.0	259 915/480 998	69.9^a^	3713/5315	51.4	445/865	36.6^a^	459/1254	89.3^a^	499/559
Smoking										
Never	55.0	263 115/478 438	47.0^a^	2479/5275	52.0	447/860	44.7^a^	557/1248	52.8	294/557
Past	34.5	165 240/478 438	40.5^a^	2137/5275	37.8	325/860	41.0^a^	512/1248	34.8	194/557
Current	10.5	50 083/478 438	12.5^a^	659/5275	10.2	88/860	14.3^a^	179/1248	12.4	69/557
Alcohol										
Daily or almost daily	20.5	98 407/479 723	14.4^a^	766/5305	16.1^a^	139/863	23.6^a^	296/1253	14.7^a^	82/558
3 or 4 times a week	23.3	111 691/479 723	16.8^a^	889/5305	22.8^a^	197/863	21.2^a^	265/1253	13.4^a^	75/558
Once or twice a week	25.9	124 130/479 723	24.7^a^	1308/5305	24.7^a^	213/863	24.8^a^	311/1253	20.4^a^	114/558
1–3 times a month	11.1	53 330/479 723	12.5^a^	661/5305	11.5^a^	99/863	9.8^a^	123/1253	15.1^a^	84/558
Special occasions only	11.4	54 443/479 723	16.6^a^	881/5305	14.1^a^	122/863	12.2^a^	153/1253	19.7^a^	111/558
Never	7.9	37 722/479 723	15.1^a^	800/5305	10.8^a^	93/863	8.4^a^	105/1253	16.7^a^	93/558
BMI	26.7 (24.1–29.8)	478 164	27.4 (24.4–31.0)^a^	5265	28.0 (25.1–31.6)^a^	861	26.9 (24.4–30.0)	1229	26.2 (23.5–30.2)	556
Quintile of Townsend index of deprivation										
1 (least deprived)	20.3	97 362/480 403	18.0^a^	957/5306	19.2	166/863	19.5	244/1253	16.7^a^	93/558
2	20.1	96 466/480 403	17.6^a^	933/5306	21.6	186/863	20.1	252/1253	17.7^a^	99/558
3	20.1	96 496/480 403	19.5^a^	1033/5306	18.4	159/863	18.6	233/1253	18.5^a^	103/558
4	20.0	95 900/480 403	20.3^a^	1078/5306	19.2	166/863	20.1	252/1253	18.8^a^	105/558
5 (most deprived)	19.6	94 179/480 403	24.6^a^	1305/5306	21.6	186/863	21.7	272/1253	28.3^a^	158/558
Medication										
Using synthetic DMARD	0.4	2050/48 0998	48.4	2574/5315	48.3	418/865	7.7	97/1254	40.4	226/559
Using biologic DMARD	0.01	66/480 998	6.2	327/5315	6.1	53/865	3.2	40/1254	0.0	0/559
Using CSs	1.1	5064/480 998	9.8	522/5315	5.1	44/865	3.8	48/1254	20.4	114/559
IPAQ group										
Low	15.4	67 394/433 680	23.0^a^	1010/4396	22.0^a^	164/745	21.4^a^	239/1115	18.6^a^	87/467
Moderate	42.2	182 781/433 680	41.2^a^	1811/4396	43.0^a^	320/745	40.1^a^	447/1115	44.5^a^	208/467
High	42.3	183 505/433 680	35.8^a^	1575/4396	35.0^a^	261/745	38.5^a^	429/1115	36.8^a^	172/467
Functional co-morbidity index										
0	49.1	235 831/480 088	37.0^a^	1962/5305	43.1^a^	372/864	42.9^a^	537/1253	38.5^a^	215/559
1–2	45.2	217 179/480 088	50.7^a^	2688/5305	47.5^a^	410/864	47.3^a^	593/1253	48.1^a^	269/559
3–4	5.2	24 786/480 088	10.8^a^	575/5305	8.8^a^	76/864	7.9^a^	99/1253	12.2^a^	68/559
≥5	0.5	2292/480 088	1.5^a^	80/5305	0.7^a^	6/864	1.9^a^	24/1253	1.3^a^	7/559

a Statistically significant difference from the none-of-the-study-conditions group (*P* < 0.05), using Mann–Whitney *U*-test for continuous variables and χ^2^ test for categorical variables. IPAQ: international physical activity questionnaire; IQR: interquartile range.

### Prevalence of co-morbidity

**Fig. 1:**
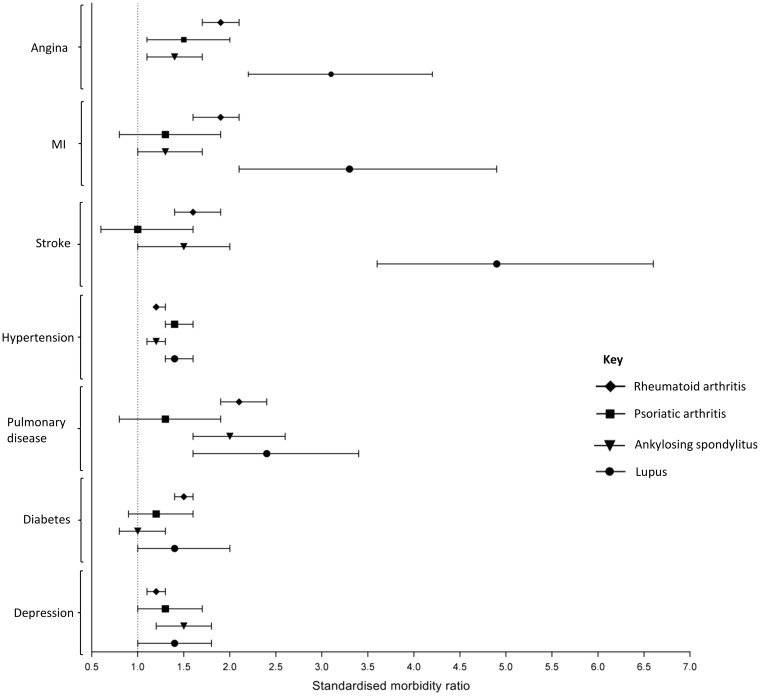
Standardized morbidity ratios for participants with a rheumatic/musculoskeletal disease Indirect age- and sex-standardized morbidity ratios for participants with a rheumatic/musculoskeletal disease. The referent group comprised participants with none of the rheumatic/musculoskeletal diseases being studied. MI: myocardial infarction.

The age- and sex-standardized prevalence of each of the co-morbidities considered was increased in at least one of the inflammatory RMDS compared with the control population (Table 2, Fig. 1). The prevalence of all co-morbidities considered was increased in participants with RA compared with controls (Table 2). In participants with SLE, the prevalence of the following co-morbidities was particularly increased: angina [SMR (95% CI): 3.1 (2.2, 4.2)], myocardial infarction [3.3 (2.1, 4.9)] and stroke [4.9 (3.6, 6.6)]. Participants with each of the inflammatory RMDs had an increased prevalence of the following co-morbidities compared with controls: angina [SMR (95% CI), RA: 1.9 (1.7, 2.1), PsA: 1.5 (1.1, 2.0), AS: 1.4 (1.1, 1.7), SLE: 3.1 (2.2, 4.2)], hypertension [RA: 1.2 (1.2, 1.3), PsA: 1.4 (1.3, 1.6), AS: 1.2 (1.1, 1.3), SLE: 1.4 (1.3, 1.6)] and depression [RA: 1.2 (1.1, 1.3), PsA: 1.3 (1.0, 1.7), AS: 1.5 (1.2, 1.8), SLE: 1.4 (1.0, 1.8)]. Only participants with RA had an increased prevalence of diabetes [1.5 (1.4, 1.6)] compared with the control population. Similar results were seen in sensitivity analysis comparing the prevalence of the co-morbidities in people who self-reported an inflammatory RMD and were also taking s/bDMARDs compared with the control population (Supplementary Table S2, available at *Rheumatology* online).

**Table 2:** Prevalence of co-morbidities in participants with a rheumatic/musculoskeletal disease

	RA		PsA		AS		SLE	
	*n* (%)	Standardized morbidity ratio^a^	*n* (%)	Standardized morbidity ratio^a^	*n* (%)	Standardized morbidity ratio^a^	*n* (%)	Standardized morbidity ratio^a^
Myocardial								
Angina	350 (0.06)	1.9 (1.7–2.1)*	42 (0.05)	1.5 (1.1–2.0)*	71 (0.05)	1.4 (1.1–1.7)*	41 (0.06)	3.1 (2.2–4.2)*
MI	224 (0.04)	1.9 (1.6–2.1)*	26 (0.03)	1.3 (0.8–1.9)	53 (0.04)	1.3 (1.0–1.7)*	23 (0.04)	3.3 (2.1–4.9)*
Vascular								
Stroke/ischaemic stroke	180 (0.03)	1.6 (1.4–1.9)*	16 (0.02)	1.0 (0.6–1.6)	40 (0.03)	1.5 (1.0–2.0)*	45 (0.07)	4.9 (3.6–6.6)*
Hypertension	2043 (36.5)	1.2 (1.2–1.3)*	345 (38.7)	1.4 (1.3–1.6)*	462 (34.8)	1.2 (1.1–1.3)*	218 (39.2)	1.4 (1.3–1.6)*
Pulmonary								
Pulmonary disease (COPD/ emphysema/bronchitis)	284 (5.1)	2.1 (1.9–2.4)*	25 (2.8)	1.3 (0.8–1.9)	63 (4.8)	2.0 (1.6–2.6)*	30 (4.7)	2.4 (1.6–3.4)*
Endocrine								
Diabetes	443 (7.9)	1.5 (1.4–1.6)*	57 (6.4)	1.2 (0.9–1.6)	84 (6.3)	1.0 (0.8–1.3)	36 (5.6)	1.4 (1.0–2.0)
Psychological								
Depression	367 (6.5)	1.2 (1.1–1.3)*	62 (7.0)	1.3 (1.0–1.7)*	94 (7.1)	1.5 (1.2–1.8)*	56 (8.7)	1.4 (1.0–1.8)*

a Age- and sex-standardized morbidity ratio. The reference population comprised participants without any of the four rheumatic/musculoskeletal diseases being studied.

* *P* < 0.05. MI: myocardial infarction; COPD: chronic obstructive pulmonary disease.

### Incidence of co-morbidities

**Fig. 2:**
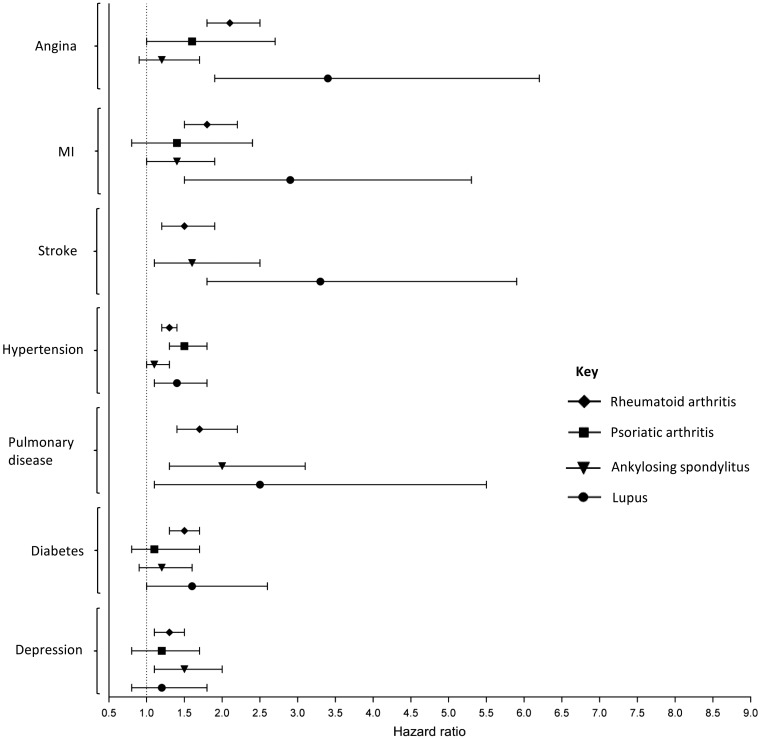
Hazard ratios for the development of co-morbidities after diagnosis of a rheumatic/musculoskeletal disease Hazard ratios calculated from a Cox proportional hazard model. Each participant with a rheumatic/musculoskeletal disease was age- and sex-matched to four participants with none of the rheumatic/musculoskeletal diseases being studied. Estimates based on less than 10 events are not presented. MI: myocardial infarction.

Most results showing a significantly higher SMR in the cross-sectional analyses investigating prevalent morbidities were also significant in the Cox regression models of incident cases of co-morbidities occurring after the diagnosis of the inflammatory RMD (Fig. 2 and Supplementary Table S3, available at *Rheumatology* online). Participants with RA were at increased risk of developing all of the co-morbidities considered, compared with controls over the same period of time. Participants with PsA had a statistically significant increased risk of developing hypertension only [hazard ratio (HR) (95% CI 1.5 (1.3, 1.8)] compared with controls. Participants with AS were at increased risk of having a stroke [HR (95% CI) 1.6 (1.1, 2.5)], developing pulmonary disease [HR (95% CI) 2.0 (1.3, 3.1)] and depression [HR (95% CI) 1.5 (1.1, 2.0)]. The risk of developing myocardial, vascular and pulmonary co-morbidities was increased in participants with SLE, with particularly increased risk of incident angina and stroke.

### Physical activity

**Fig. 3:**
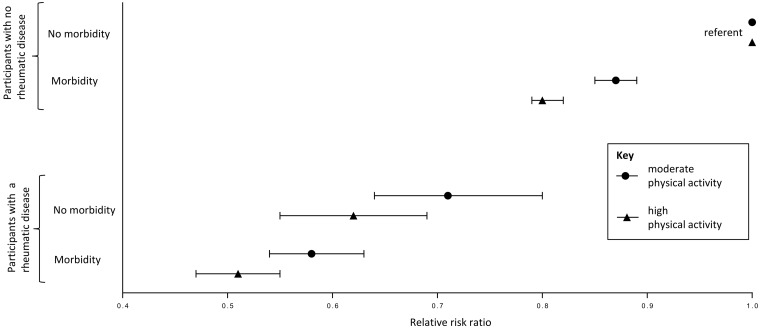
Association between presence/absence of rheumatic/musculoskeletal disease, (co)morbidity and physical activity Results from multinomial logistic regression. Physical activity group (referent = low) is the dependent variable. Study group: no rheumatic/musculoskeletal disease and no morbidity (no RD-noM), no rheumatic/musculoskeletal disease and morbidity (no RD-M), rheumatic/musculoskeletal disease and no co-morbidity (RD-no M), and rheumatic/musculoskeletal disease and co-morbidity (RD-M) is the independent variable. Adjusted for age, sex, smoking, alcohol consumption, BMI and Townsend deprivation index.

A significantly lower proportion of people with one of the four inflammatory RMDs reported performing a high level of physical activity, compared with the control population (Table 1). The proportion of participants meeting the WHO recommended amount of physical activity was 64% for people with an inflammatory RMD and 74% for people without an inflammatory RMD (*P *< 0.001, χ^2^ test). The presence of (co)morbidity was associated with reduced odds of reporting a moderate or high level of physical activity in participants with an inflammatory RMD and in participants without an inflammatory RMD, with low physical activity as the referent group (Fig. 3). Compared with participants with no inflammatory RMD and no morbidity and low level of physical activity (referent), participants with no inflammatory RMD and a morbidity were less likely to report a moderate [OR (95% CI), 0.87 (0.85, 0.89)] or high [0.80 (0.79, 0.82)] level of physical activity (Table 3, Fig. 3). Compared with the referent group, participants with an inflammatory RMD and no morbidity were less likely to report a moderate [OR (95% CI), 0.72 (0.64, 0.80)] or high [0.61 (0.55, 0.69)] level of physical activity, and participants with an inflammatory RMD and a morbidity were even less likely to report a moderate [OR (95% CI), 0.58 (0.54, 0.63)] or high [0.51 (0.47, 0.55)] level of physical activity (Fig. 3).

**Table 3:** Association between co-morbidity and physical activity in participants with a rheumatic/musculoskeletal disease

	Physical activity level	
	RRR (95% CI)^a^	
	Moderate	High
Myocardial		
Angina	0.60 (0.46, 0.78)*	0.54 (0.41, 0.71)*
MI/heart attack	0.68 (0.50, 0.92)*	0.54 (0.39, 0.75)*
Vascular		
Stroke/ischaemic stroke	0.55 (0.39, 0.78)*	0.65 (0.46, 0.92)*
Hypertension	0.75 (0.66, 0.86)*	0.71 (0.62, 0.82)*
Pulmonary		
Pulmonary disease (COPD/emphysema/bronchitis)	0.86 (0.64, 1.16)	0.72 (0.53, 0.99)*
Psychological		
Depression	0.67 (0.52, 0.85)*	0.77 (0.60, 0.98)*
Functional co-morbidity index		
0	referent	referent
1–2	0.72 (0.63, 0.83)*	0.68 (0.59, 0.78)*
3–4	0.48 (0.38, 0.60)*	0.48 (0.38, 0.60)*
≥5	0.32 (0.20, 0.54)*	0.22 (0.13, 0.40)*

a Relative risk ratio from a multinomial logistic model with physical activity level as the dependent group. Low physical activity was the referent group. Adjusted for age and sex.

* *P* < 0.05. RRR: relative risk ratio; MI: myocardial infarction; COPD: chronic obstructive pulmonary disease.

In people with one of the four inflammatory RMDs, most of the co-morbidities considered were individually associated with physical activity level (Table 3). In particular, myocardial and vascular co-morbidities and depression were associated with reduced odds of reporting a moderate or high level of physical activity. There was evidence of a relationship between increasing level of co-morbid burden, measured using a modified functional co-morbidity index, and reduced odds of reporting a moderate or high level of physical activity (Table 3).

## Discussion

In this large national UK cohort we found an increased prevalence and incidence of chronic myocardial, vascular and pulmonary co-morbidities and depression in people with a range of chronic inflammatory RMDs compared with those without these conditions. Participants with an inflammatory RMD were less likely to perform a moderate or high level of physical activity compared with those without these inflammatory RMDs. Myocardial and vascular co-morbidities, and depression, as well as an increased level of co-morbid burden, were associated with reduced odds of having a moderate or high level of physical activity in participants with an inflammatory RMD.

Our results are similar to previous cross-sectional studies, showing an increased prevalence of chronic co-morbidities in people with an inflammatory RMD. Data from the Netherlands Information Network of General Practice showed an increased prevalence of chronic obstructive pulmonary disease (40% increase), cardiovascular disease (40% increase) and depression (20% increase) at the time of diagnosis of inflammatory arthritis compared with age- and sex-matched controls [7]. Similar results have been found in people with AS [13], PsA [12] and RA [9, 11].

Two previous meta-analyses showed that patients with RA have almost a 2-fold risk of developing chronic obstructive pulmonary disease [33], and a 70% increased risk of having a myocardial infarction compared with controls [34]. Data from the Dutch Primary Care Database has also showed that patients with inflammatory arthritis have a 40% increased risk of developing depression compared with controls without inflammatory arthritis [8].

There is a paucity of data comparing the prevalence of co-morbidities across different inflammatory RMDs. A cross-sectional analysis of medical service and prescription drug claims database from the USA, found a 30% increased prevalence of hypertension in people with either RA, PsA or AS, compared with controls without any of these conditions [11], which is in line with our findings.

Our results are consistent with other studies, showing that people with RMDs are less physically active compared with the general population [21], and that a significant proportion do not carry out the recommended level of physical activity [35, 36]. Two previous studies did not see an association between co-morbidity and physical activity in people with a RMD [37]. The discordance between our results and those studies may be explained by differences in the study population: subjects included in the previous two studies also had other forms of RMD, including OA and gout.

The increased prevalence of chronic co-morbid diseases seen in participants with an inflammatory RMD compared with participants without an inflammatory RMD may, in part, be explained by the increased prevalence of traditional risk factors for disease, such as smoking. Further work is needed to determine how lifestyle and environmental, genetic and epigenetic risk factors, as well as the independent effect of the inflammatory RMD and treatment, contribute to the increased risk of chronic co-morbid diseases.

Our study has a number of strengths. The most prevalent chronic inflammatory RMDs were studied in a single large national cohort with detailed demographic and lifestyle data, and with details about chronic diseases and medication collected in a consistent way. There are also some limitations to our study. Due to the self-reported nature of chronic disease and physical activity data, there is the potential for misclassification. However, the prevalence of inflammatory RMD in the UK Biobank match closely with previously published estimates [3, 38]. There has been limited validation of self-reported medical conditions in the UK Biobank to date; however, one study has suggested that the prevalence of overall pain and musculoskeletal-specific pain in the UK Biobank closely match estimates from large population studies with much higher participation rates [39]. Some participants reported being diagnosed with an inflammatory RMD before the age of 18: 246 (4.6%), 20 (2.3%), 71 (5.7%) and 11 (2.0%) for RA, PsA, AS and SLE, respectively. These subjects were included in the present study; they may represent patients with juvenile arthritis that has persisted into adulthood. Because data on physical activity were collected at a single point in time, it was not possible to determine the temporal relationship between physical activity and co-morbidity. Co-morbid conditions may impact on the ability of people with an inflammatory RMD to carry out physical activity; however, it is also possible that people with a historically low level of physical activity may be more likely to develop co-morbid conditions. Data on disease activity was not available. However, of six studies that have looked at the association between disease activity and physical activity, only one found a modest association [40]. The response rate for the UK Biobank was 5.5%. Nonetheless, while the UK Biobank cohort may not necessarily be representative of the whole UK population in all respects, internal comparisons of the rates of co-morbidities between people with and without an inflammatory RMD, and internal analysis of the association between co-morbidity and physical activity is valid, given the sufficiently large number of people with different exposures included in this cohort [39, 41, 42].

In conclusion, patients with chronic inflammatory RMDs have an increased risk of developing chronic co-morbidities compared with the general population. Early detection and optimal management of co-morbid conditions in patients with an inflammatory RMD may help to reduce the impact of the increased co-morbid burden seen in these patients. Patients with an inflammatory RMD should be encouraged to meet physical activity guidelines where possible, which may help to reduce the risk of incident cardiovascular disease. Furthermore, co-morbidity should be taken into account in studies looking at physical activity in patients with inflammatory RMDs.

## Supplementary Material

Supplementary Data
