# Supplementary material for: The prevalence of co-morbidities and their impact on physical activity in people with inflammatory rheumatic diseases compared with the general population: results from the UK Biobank
Source: Rheumatology (Oxford). 2018 Aug 9;57(12):2172–82. doi: 10.1093/rheumatology/key224 (PMC6256331; doi:10.1093/rheumatology/key224)
Supplement: Supplementary Data [file key224_suppl_data.docx]

**SUPPLEMENTARY DATA**

**METHODS**

**Coding free text medication**

Medication used to treat rheumatic and musculoskeletal diseases was identified from the musculoskeletal chapter of the British National Formulary. Spelling errors in drug names recorded as free text during the interview with a research nurse were corrected using the spellchecking library PyEnchant for Python (<http://pythonhosted.org/pyenchant/>).  PyEnchant checks the spelling of words by matching the free text drug names to a drug dictionary and gives suggestions for misspelled words. The drug dictionary that was used in the current study was downloaded from Drungbank ([www.drungbank.ca](http://www.drungbank.ca)) which is a unique resource containing detailed drug data. Further details of the process used to code free text medication data are available from the corresponding author on request.

**Construction of the functional comorbidity index**

The functional comorbidity index is calculated as a sum of the following comorbidities present: osteoporosis, asthma, chronic obstructive pulmonary disease (COPD), angina, congestive heart failure/ heart disease, heart attack, neurological disease, stroke, peripheral vascular disease, diabetes (types I and II), upper gastrointestinal disease, depression, anxiety or panic disorders, visual impairment, hearing impairment, degenerative disc disease.

**Supplementary table S1. Differences in subject characteristics between participants included and excluded from analyses**

|  | Subjects include in the study (*N*=488,991) | | Subjects excluded from the study (*N*=13,652) | |
| --- | --- | --- | --- | --- |
|  | Mean (SD) or % | *n* | Mean (SD) or % | *n* |
| Age | 56·5 (8·1) | 488991 | 57·9 (7·9) | 13652 |
| Female | 54·2 | 265031/488991 | 61·8 | 8436/13652 |
| Smoking |  |  |  |  |
| never | 54·9 | 266892/486378 | 49·9 | 6663/13366 |
| past | 34·6 | 168408/486378 | 35·9 | 4792/13366 |
| current | 10·5 | 51078/486378 | 14·3 | 1911/13366 |
| Alcohol |  |  |  |  |
| daily or almost daily | 20·4 | 99690/487702 | 15·6 | 2101/13438 |
| three or four times a week | 23·2 | 113117/487702 | 17·5 | 2345/13438 |
| once or twice a week | 25·9 | 126076/487702 | 24·2 | 3246/13438 |
| one to three times a month | 11·1 | 54297/487702 | 11·7 | 1577/13438 |
| special occasions only | 11·4 | 55709/487702 | 17·3 | 2321/13438 |
| never | 8·0 | 38813/487702 | 13·8 | 1846/13438 |
| BMI | 27·4 (4·8) | 486075 | 28·9 | 13463 |
| Quintile of Townsend index of deprivation |  |  |  |  |
| 1 (least deprived) |  |  |  |  |
| 2 |  |  |  |  |
| 3 |  |  |  |  |
| 4 |  |  |  |  |
| 5 (most deprived) |  |  |  |  |
| IPAQ group |  |  |  |  |
| *low* | 15·6 | 68894/440403 | 16·7 | 1792/10757 |
| *moderate* | 42·1 | 185567/440403 | 42·8 | 4603/10757 |
| *high* | 42·2 | 185942/440403 | 40·6 | 4362/10757 |
| Functional index |  |  |  |  |
| *0* | 49·0 | 238917/488069 | 33.6 | 1319/3922 |
| *1-2* | 45·3 | 221139/488069 | 53.6 | 2103/3922 |
| *3-4* | 5.3 | 25604/488069 | 11.2 | 440/3922 |
| *≥5* | 0·5 | 2409/488069 | 1.5 | 60/3922 |

Statistically significantly differences were found between participants included and excluded from analyses for all variables (p<0.0001), using Mann-Whitney U-test for continuous variables and chi-square test for categorical variables. IPAQ: international physical activity questionnaire.

**Supplementary table S2. Prevalence of comorbidities in participants with a rheumatic/musculoskeletal disease (self-reported a rheumatic/musculoskeletal disease and also using a DMARD)**

|  | Rheumatoid arthritis | | Psoriatic arthritis | | Ankylosing spondylitis | | Lupus | |
| --- | --- | --- | --- | --- | --- | --- | --- | --- |
|  | n (%) | Standardised morbidity ratio^a^ | n (%) | Standardised morbidity ratio^a^ | n (%) | Standardised morbidity ratio^a^ | n (%) | Standardised morbidity ratio^a^ |
| **Myocardial** |  |  |  |  |  |  |  |  |
| Angina | 134 (0.05) | 1.5 (1.3, 1.8)* | 21 (0.04) | 1.6 (1.0, 2.4) | 8 (0.07) | 1.9 (0.8, 3.8) |  | 5.5 (1.3, 4.0)* |
| MI | 103 (0.04) | 1.8 (1.5, 2.2)* | 12 (0.02) | 1.2 (0.6, 2.1) | -^b^ | -^b^ |  | 2.0 (0.7, 4.4) |
| **Vascular** |  |  |  |  |  |  |  |  |
| Stroke / Ischaemic stroke | 73 (0.03) | 1.4 (1.1, 1.8)* | -^b^ | -^b^ | -^b^ | -^b^ | 17 (0.06) | 4.4 (2.6, 7.1)* |
| Hypertension | 967 (0.4) | 1.2 (1.2, 1.3)* | 176 (0.4) | 1.5 (1.3, 1.7)* | 60 (0.5) | 1.8 (1.4, 2.3)* | 93 (0.3) | 1.4 (1.2, 1.8)* |
| **Pulmonary** |  |  |  |  |  |  |  |  |
| Pulmonary disease (COPD/emphysema/bronchitis) | 133 (0.05) | 2.1 (1.7, 2.4)* | -^b^ | -^b^ | -^b^ | -^b^ | 16 (0.06) | 2.9 (1.7, 4.8)* |
| **Endocrine** |  |  |  |  |  |  |  |  |
| Diabetes | 188 (0.07) | 1.3 (1.2, 1.5)* | 27 (0.06) | 1.2 (0.8, 1.7) | 13 (0.1) | 1.9 (1.0, 3.2)* | 14 (0.05) | 1.3 (0.7, 2.1) |
| **Psychological** |  |  |  |  |  |  |  |  |
| Depression | 145 (0.05) | 1.0 (0.8, 1.1) | 37 (0.08) | 1.6 (1.1, 2.2)* | 15 (0.1) | 2.5 (1.4, 4.2)* | 25 (0.09) | 1.4 (0.9, 2.0) |

^a^Age- and sex-standardised morbidity ratio. The reference population comprised participants without any of the four rheumatic/musculoskeletal diseases being studied. ^b^Results are not presented where the number of cases is <10. MI: myocardial infarction; COPD: chronic obstructive pulmonary disease

**Supplementary table S3. Comorbidities developing after the diagnosis of rheumatic/musculoskeletal disease**

|  | Rheumatoid arthritis | | | Psoriatic arthritis | | | Ankylosing spondylitis | | | Lupus | | |
| --- | --- | --- | --- | --- | --- | --- | --- | --- | --- | --- | --- | --- |
|  | Cases, n (%) | Controls^a^, n (%) | HR^b^ (95% CI) | Cases, n (%) | Controls^a^, n (%) | HR^b^ (95% CI) | Cases, n (%) | Controls^a^, n (%) | HR^b^ (95% CI) | Cases, n (%) | Controls^a^, n (%) | HR^b^ (95% CI) |
|  | (*N*=5315) | (*N*=21260) |  | (*N*=865) | (*N*=3460) |  | (*N*=1254) | (*N*=5016) |  | (*N*=559) | (*N*=2236) |  |
| **Myocardial** |  |  |  |  |  |  |  |  |  |  |  |  |
| Angina | 195 (3.7) | 377 (1.8) | 2.1 (1.8, 2.5)* | 22 (2.5) | 54 (1.6) | 1.6 (1.0, 2.7) | 52 (4.2) | 169 (3.4) | 1.2 (0.9, 1.7) | 20 (3.6) | 24 (1.1) | 3.4 (1.9, 6.2)* |
| MI | 122 (2.3) | 273 (1.3) | 1.8 (1.5, 2.2)* | 17 (2.0) | 49 (1.4) | 1.4 (0.8, 2.4) | 44 (3.5) | 129 (2.6) | 1.4 (1.0, 1.9) | 14 (2.5) | 19 (0.8) | 2.9 (1.5, 5.3)* |
| **Vascular** |  |  |  |  |  |  |  |  |  |  |  |  |
| Stroke / Ischaemic stroke | 95 (1.8) | 247 (1.2) | 1.5 (1.2, 1.9)* | -^c^ | 32 (0.9) | -^c^ | 31 (2.5) | 77 (1.5) | 1.6 (1.1, 2.5)* | 21 (3.8) | 25 (1.2) | 3.3 (1.8, 5.9)* |
| Hypertension | 1076 (20.2) | 3317 (15.6) | 1.3 (1.2, 1.4)* | 190 (22.0) | 521 (15.1) | 1.5 (1.3, 1.8)* | 301 (24.0) | 1068 (21.3) | 1.1 (1.0, 1.3) | 96 (17.2) | 278 (12.4) | 1.4 (1.1, 1.8)* |
| **Pulmonary** |  |  |  |  |  |  |  |  |  |  |  |  |
| Pulmonary disease (COPD/emphysema /bronchitis) | 103 (1.9) | 236 (1.1) | 1.7 (1.4, 2.2)* | -^c^ | 30 (0.9) | -^c^ | 31 (2.5) | 62 (1.2) | 2.0 (1.3, 3.1)* | 10 (1.8) | 17 (0.8) | 2.5 (1.1, 5.5)* |
| **Endocrine** |  |  |  |  |  |  |  |  |  |  |  |  |
| Diabetes | 248 (4.7) | 665 (3.1) | 1.5 (1.3, 1.7)* | 32 (3.7) | 112 (3.2) | 1.1 (0.8, 1.7) | 63 (5.0) | 203 (4.1) | 1.2 (0.9, 1.6) | 22 (3.5) | 54 (2.1) | 1.6 (1.0, 2.6) |
| **Psychological** |  |  |  |  |  |  |  |  |  |  |  |  |
| Depression | 196 (3.7) | 540 (2.5) | 1.3 (1.1, 1.5)* | 32 (3.7) | 101 (2.9) | 1.2 (0.8, 1.7) | 64 (5.1) | 159 (3.2) | 1.5 (1.1, 2.0)* | 34 (5.4) | 97 (3.8) | 1.2 (0.8, 1.8) |

^a^Each case was age- and sex-matched to controls from participants who do not have any of the rheumatic diseases being studied. ^b^Hazard ratio from Cox proportional hazard model. Each participant with a rheumatic disease was age- and sex-matched to 4 controls with none of the rheumatic diseases being studied. ^c^Estimates based on less than 10 events are not presented. *p<0.05. MI: myocardial infarction; COPD: chronic obstructive pulmonary disease.

**Supplementary Figure 1.** Participant flow diagram

502,643

missing data for medical conditions 9720

>1 rheumatic/musculoskeletal disease 137

nonspecified arthritis 3795

488,991

Control

480,998

RA

5,315

PsA

865

AS

1,254

SLE

559
